# Failure of current Alzheimer’s disease hypotheses

**DOI:** 10.18632/aging.204880

**Published:** 2023-07-01

**Authors:** Paul L. Wood

**Affiliations:** 1 Metabolomics Unit, College of Veterinary Medicine, Lincoln Memorial University, Harrogate, TN 37752, USA

**Keywords:** Alzheimer’s disease, omics, cholinergic, amyloid, neuroinflammation

Defining the etiology of cognitive decline in Alzheimer’s disease (AD) has proven to be a monumental task with massive resources having limited success to-date. The current hypotheses include cholinergic dysfunction, amyloid deposition, abnormal neuroinflammation, and white matter dysfunction. Classical biochemical methods first identified decrements in the cortical cholinergic synthetic enzyme choline acetyltransferase which resulted in the successful clinical development of cholinesterase inhibitors to potentiate cholinergic neurotransmission in AD [1]. However, continued decline of cholinergic function is not altered with these inhibitors, resulting in a limited time window of clinical utility.

Next the amyloid hypothesis suggested that excessive amyloid deposition is responsible for neuronal dysfunction and that inhibitors of this process would halt disease progression. This has not proven to be the case with lecanemab, the only approved drug in this area, demonstrating limited efficacy in early-onset patients and the risk of microbleeds [2]. In addition, the amyloid hypothesis does not take into account the significant number of elderly Non-Demented individuals with Alzheimer's Disease Neuropathology (NDAN) [3].

The neuroinflammatory hypothesis is extremely complex in that it is hypothesized to involve a variety of mediators [4, 5]. These include inflammatory cytokines and prostaglandins, produced by microglia, which are responsible for “by-stander neuronal lysis”. In addition, increased production of reactive aldehydes and nitric oxide are hypothesized to modify neuronal protein function, via covalent linkages. To-date the only clinical trials to test the neuroinflammatory hypothesis have been with cyclooxygenase inhibitors, which failed in prospective trials.

Data obtained with imaging of white matter hyperintensities have been the foundation of the hypothesis of white matter dysfunction in AD [6]. Lipidomics studies have not found altered white matter lipid composition in AD subjects [7], supporting suggestions that white matter hyperintensities may be biomarkers of cerebral microbleeds/vascular disease.

Both academia and the pharmaceutical sector have invested heavily in the cholinergic, amyloid, and neuroinflammatory hypotheses with limited return. Current hypotheses are insufficient to explain the pathogenesis of cognitive deficits in AD. Therefore, what are the next critical steps to advance research in this field. The omics technologies have not provided data that points in a new direction. Genomics has identified risk factors for AD but not gene defects essential for the decrements in cognitive function characteristic of AD. Similarly, despite large decreases in brain volume in AD, proteomics, metabolomics, and lipidomics have not identified alterations essential to the advancement of AD cognitive deficits. One potential area of research that might yield the answers we seek involves investigations of NDAN subjects who remain cognitively intact despite significant AD brain neuropathology. Neuronal shrinkage is thought to account for reductions in grey matter and the decline in cognitive abilities, associated with aging. However, brain shrinkage also is present in NDAN cases. It is essential to understand the resistance mechanisms involved in the protection of cognition in NDAN subjects [3, 8].

It is therefore of high interest to define the mechanisms that provide the capacity of NDAN subjects to evade cognitive decline/dysfunction. One suggestion that has been published is that microglial function in NDAN subjects is more efficient in removing damaged synapses than in AD subjects [8]. This hypothesis highlights the need to determine if cholinergic function is normal in NDAN subjects since this has not been investigated to-date.

In summary, we are at a new crossroad in AD research. New priorities for funding strategies urgently need to be established and tough decisions will need to be made by the NIH and by biotech and pharmaceutical companies.
